# Physical performance and tactical experience: determinants of competition outcomes in amateur vs. elite cyclists – a descriptive study from the UCI tour of Lithuania

**DOI:** 10.3389/fphys.2026.1768076

**Published:** 2026-02-12

**Authors:** Leonardo Cesanelli, Daniele Conte, Berta Ylaite, Bent R. Rønnestad, Thomas Lagoute, Tomas Venckunas, Fabio Zambolin

**Affiliations:** 1 Institute of Sport Science and Innovations, Lithuanian Sports University, Kaunas, Lithuania; 2 Department of Movement, Human and Health Sciences, University of Rome “Foro Italico”, Rome, Italy; 3 Department of Coaching Science, Lithuanian Sports University, Kaunas, Lithuania; 4 Institute of Mechatronics, Kaunas University of Technology, Kaunas, Lithuania; 5 Section for Health and Exercise Physiology, Inland Norway University of Applied Sciences, Lillehammer, Norway; 6 Department of Sport Science and Physical Education, University of Agder, Kristiansand, Norway; 7 Department of Sport and Exercise Sciences. Manchester Metropolitan University, Manchester, United Kingdom; 8 Manchester Metropolitan University Institute of Sport, Manchester Metropolitan University, Manchester, United Kingdom

**Keywords:** critical power, cycling performance, endurance, power distribution, tactical behavior

## Abstract

**Introduction:**

The aim of this descriptive study was to compare laboratory and in-competition performance metrics between elite and amateur cyclists competing in the 2024 UCI Tour of Lithuania with respect to physical performance, racing approach, and tactical behavior.

**Methods:**

Two teams of five elite and five amateur cyclists completed laboratory tests 1 month before the event. Tests assessed peak oxygen uptake ( ${\dot{V}O}_{2\text{peak}}$ ), gross efficiency, peak power in incremental and sprint protocols, and seasonal power records. During the five-stage race (870.2-km, 4,039-m elevation gain), competition data, including stage and general classification results, power data, heart rate, and gpx tracks, were analyzed. Descriptive statistics and Cohen’s d effect sizes (ES) were used to assess between-group differences.

**Results:**

Amateurs exhibited lower ${\dot{V}O}_{2\text{peak}}$ , maximal aerobic and critical power (ES: 1.68–2.37). During competition, amateurs spent more time above critical power (ES: 2.09–3.25) and exhibited a more homogeneous power distribution within their team (ES: 0.49–1.82). In contrast, elite cyclists displayed a polarized effort profile. These differences corresponded to poorer stage and overall classification results for amateurs (ES: 1.39), while elites followed specific competition strategies, contributing to better stage and overall results. Nevertheless, all amateurs successfully completed the five-stage race.

**Discussion:**

Amateur cyclists demonstrated sufficient physical capacity to complete the Tour of Lithuania despite lower aerobic capacity and limited experience in team tactics. These findings illustrate the rising performance levels in amateur cycling while emphasizing persistent gaps in physical conditioning, race experience and execution, particularly in energy management, pacing strategies, and situational decision-making, factors that remain key competition-specific performance limitations compared to elite cyclists.

## 1 Introduction

Over the past three decades, cycling performance optimization has steadily advanced, driven by scientific, technological, material, and logistical innovations (Phillips and Hopkins, 2020). These developments have significantly influenced performance trajectories, competition strategies, and the overall level of competition (Alejo et al., 2022; Almquist et al., 2023; Cesanelli et al., 2024). Notably, such advancements have not been limited to elite athletes but have also permeated the amateur cycling community (Cesanelli et al., 2023; Cesanelli et al., 2024). This has led to a growing interest in competitive cycling among non-professional athletes, with many striving to compete at increasingly higher levels (Ferrucci et al., 2021; Ferrucci et al., 2022).

Several studies have sought to understand this phenomenon, comparing elite cyclists—who dedicate their lives to professional training and achieving peak performance—with competitive amateur cyclists, who pursue their maximum potential alongside their regular jobs and daily responsibilities (Petersen et al., 2017; Priego Quesada et al., 2018; Martínez-Noguera et al., 2021; Cesanelli et al., 2023; Cesanelli et al., 2024). Results suggest that top-level amateur cyclists may compete with elite cyclists in second-tier competitions where direct comparisons are possible (Cesanelli et al., 2024). However, lower physical performance metrics and, more critically, a lack of high-level competition experience and abilities (e.g., energy management during races) appear to be significant barriers to successfully challenging professional athletes (Cesanelli et al., 2024). Despite some indications, the primary factors limiting comparable competition outcomes between these groups remain unclear.

To address this, we analyzed and described a specific competition scenario involving an elite and an amateur team participating in the 2024 UCI Tour of Lithuania (TOL). This stage race allowed Lithuania’s top amateur team to compete alongside professional cyclists. In this descriptive study both laboratory performance data and in-field competition metrics are presented, focusing on physical performance and tactical behavior.

## 2 Materials and methods

### 2.1 Participants

Five elite cyclists (23.4 ± 2.3 years, 78.0 ± 5.8 kg, 183 ± 3 cm, cycling 23,539 ± 5,430 km per year) and five competitive-level amateur cyclists (36.4 ± 3.7 years, 85.7 ± 4.8 kg, 187 ± 5 cm, cycling 12,801 ± 6,073 km per year) voluntarily participated in this study which was conducted between 20/01/2024 and 08/06/2024. Written informed consent was obtained from all participants prior to enrollment. Elite cyclists were full-time professionals with at least 3 years of UCI racing experience, training 14–28 h/week depending on the season (∼45 days of racing in 2024). Amateurs had no structured youth training in cycling, taking up the sport at age 26 or later and progressively competing at a high level non-professionally, with training volumes of 6–15 h/week (∼15 days of racing in 2024). Both groups targeted the national championships as their main season goal, ensuring similar training focus leading up to the TOL. The research protocol adhered to the principles of the Declaration of Helsinki and received formal approval from the Lithuanian Sports University Ethics Committee (MNL-SVA (M)-2023-578). The sample size was determined *a priori* by the fixed roster composition of the two teams participating in the Tour of Lithuania, which made a conventional power-based estimation infeasible.

### 2.2 Experimental design

Two cycling teams (five cyclists per team) registered for the 2024 Tour of Lithuania (5 stages, 870.2 km, 4,039 vertical m; https://touroflithuania.eu) were tested prior to and monitored during the competition. Laboratory tests were conducted 1 month before the event to assess the athletes’ physical performance. Data on competition results, power output, heart rate (HR), GPX tracks, and stage video-analysis (from live streaming records), were collected and analyzed overall and from each stage.

### 2.3 Testing procedures

Following anamnestic interviews and anthropometric measurements, cyclists underwent two separate performance assessments, scheduled 1 week apart: a maximal incremental exercise test on the first day and a cycling efficiency test followed by a 6-s sprint on the second day.

Upon arrival at the laboratory, body mass (Tanita-305 body-fat analyzer, Tanita Corp.) and body height (PERSON-CHECK Stadiometer, KaWe) were recorded. All tests were conducted in a controlled environment (20 °C–22 °C, 50%–60% relative humidity). For ${\dot{V}O}_{2\text{peak}}$ determination, participants completed a 5-min warm-up at 120 W, followed by an incremental ramp test (+20 W/min) on an electromagnetically braked ergometer (Lode Excalibur Sport, Lode BV) (Pettitt et al., 2013; Bayles, 2023). Power (W) and cadence (rpm) were continuously recorded, while cadence was self-selected, and the ergometer setup was individually adjusted. Gas exchange was analyzed in breath-by-breath mode (MetaLyzer 3B, Cortex Biophysik), calibrated before each test. HR was continuously monitored (Polar H10, Polar Electro). The test was terminated if cadence dropped by > 10 rpm for >10 s despite strong verbal encouragement. ${\dot{V}O}_{2\text{peak}}$ was determined as the highest 30-s average ${\dot{V}O}_{2}$ value, while peak power output (PPO) was identified as the average of power sustained for the last 60-s of the incremental test. Both variables were expressed in absolute and relative terms.

On a separate day, cycling gross efficiency (GE) was assessed using the MetaLyzer 3B and the Excalibur Sport ergometer. Following a 4-min warm-up (free pedaling from 80 W to the target load), cyclists pedaled for 5 min at 45% PPO, maintaining 75 ± 2 rpm cadence (Hopker et al., 2009; MacDougall et al., 2022). GE (%) was calculated as: $\frac{Total\;work}{Energy\;expended}\times 100$ (Hopker et al., 2009; MacDougall et al., 2022). Valid GE data required RER <1, excluding the first minute. Pedaling technique was analyzed via crank torque data, using positive impulse percentage (PIP) as a marker (García-López et al., 2016; Cesanelli et al., 2024). After a 60-min rest, participants completed a 10-s sprint test with a resistance of 0.75 N/kg body mass, preceded by two familiarization trials (Cesanelli et al., 2024).

### 2.4 Competition data

Official competition results and power output data from cyclists calibrated personal power meters (Wahoo Speedplay power, Rotor INSPIDER, Favero Assioma, and Garmin Vector 2) were analyzed using GoldenCheetah (v3.6) (Cesanelli et al., 2023; Cesanelli et al., 2024). All power meters were zero-offset and calibrated before each training session and competition in accordance with manufacturer instructions. As previously described (Valenzuela et al., 2022) each participant’s competition and seasonal (competitions and training data from March to October) power profile and critical power (CP) were established based on their highest attained maximum mean power values across multiple effort durations. CP was estimated at the individual level using a two-parameter critical power model (hyperbolic power–duration relationship), as implemented in GoldenCheetah, v3.6, with CP and W′ derived from the linearized work–time relationship. The CP model fitting included best-effort durations ranging from 2 to 180 min. Only durations with valid maximal mean power values were included in the fitting procedure. Outlier efforts (e.g., isolated unusually high values inconsistent with the individual’s overall power–duration profile) were identified through visual inspection of power–duration plots and excluded when clearly attributable to recording artifacts or non-representative events. Missing durations did not preclude CP estimation provided sufficient data points were available across the selected time range. Additionally, average power over each of the full competition, W′ work, training stress score (TSS), and external energy expenditure (EE) were calculated (Poole et al., 2016; Cesanelli et al., 2023; Cesanelli et al., 2024). Power data were visually and algorithmically screened to identify potential anomalies, including unrealistically high power spikes, abrupt signal dropouts, and cadence–power inconsistencies. Files presenting clear recording artifacts (e.g., single-point spikes not supported by cadence or duration, or prolonged signal loss) were excluded from analysis or trimmed accordingly. CP and all derived variables were computed individually for each cyclist and subsequently averaged at the group level for descriptive comparison. All analyses were performed on individual datasets using each cyclist’s own power meter, and values were subsequently aggregated at the group level. While the use of different power meter models may introduce minor inter-device variability, this approach reflects real-world competition conditions and was considered acceptable for descriptive comparisons. A dedicated open-source software (GoldenCheetah, v3.6) was employed for all analyses.

Additionally, comprehensive video analysis of the five TOL stages was conducted by two researchers (L.C. and B.Y.) using live-stream recordings from the official race website. This qualitative analysis focused on team tactics by observing rider actions and race conduct in relation to assigned roles (e.g., sprinter, leader, domestique), if any, and linking these behaviors to final race outcomes and performance metrics.

A predefined coding framework was applied, in which tactical behaviors were categorized *a priori* based on existing literature and practical race analysis. Coded behaviors included rider positioning within the peloton (front, mid, rear), drafting behavior (prolonged sheltered riding vs. exposed riding), participation in lead-out trains, response to breakaways (active chase, passive following, or no response), and protective actions toward a designated team leader or sprinter. Operational definitions were agreed upon before analysis to ensure consistency. Both raters were experienced in cycling performance analysis and were not blinded to team identity due to the nature of race footage and team kits; however, coding was performed independently, and discrepancies were resolved through discussion. Inter-rater agreement for tactical behavior classification was high (percentage agreement >85%; disagreements were resolved by consensus following discussion and, when required, consultation with a third researcher). For example, elite team strategies such as protecting the sprinter in sprint finishes or shielding the team leader during key race phases were identified and interpreted in light of power data and stage results. These events were typically associated with distinct power profiles, such as prolonged sub-CP riding followed by short supra-CP efforts during decisive race moments (e.g., lead-outs or positioning before final kilometers). For amateurs, rider positioning and group dynamics were tracked to infer race behavior and contextualize their pacing and performance profiles, with frequent exposure to wind and repeated supra-CP efforts reflecting less structured drafting and positioning strategies. Overall, this analysis provided a qualitative narrative of competition dynamics, race strategies, and tactical decisions. Power data were further used to characterize effort distribution across different race phases, including time spent above CP and the percentage of power above or below each stage’s mean power. These insights, combined with video observations, enabled a detailed description of each cyclist’s race actions, identifying their tactical role (e.g., domestique, sprinter, leader) and the strategic approach within each stage and the overall competition.

### 2.5 Statistical analysis

All analyses were conducted using IBM SPSS Statistics for Windows® (version 28.0.0.0). Given the relatively small sample size (two teams participating in the TOL, n = 5 per group), descriptive statistics (mean ± SD) were reported. To provide insight into the magnitude of between-group differences, standardized mean differences were calculated using Cohen’s d. Effect sizes (ES) were interpreted as follows: trivial (T), <0.20; small (S), 0.20–0.59; moderate (M), 0.60–1.19; large (L), 1.20–1.99; and very large (V), ≥2.00 (Fritz et al., 2012).

## 3 Results

The main laboratory variables assessed for physical performance evaluation are presented in Table 1. Elite cyclists exhibited higher ${\dot{V}O}_{2\text{peak}}$ and power metrics (PPO, sprint PP), particularly when normalized to body mass.

**TABLE 1:** Physical performance variables assessed from laboratory tests (i.e., maximal incremental test, gross efficiency test and sprint test).

Performance variable	Elite (*n* = 5)	Amateurs (*n* = 5)	ES (95% CI)
${\dot{V}O}_{2\text{peak}}$ (L)	5.01 ± 0.32	4.61 ± 0.11	1.68 (0.19–3.17)
${\dot{V}O}_{2\text{peak}}$ (mL/min/kg)	64.41 ± 5.56	53.86 ± 2.93	2.37 (0.67–4.07)
PPO (W)	466.19 ± 17.57	428.56 ± 22.33	1.87 (0.33–3.41)
PPO (W/kg)	6.01 ± 0.63	5.01 ± 0.17	2.17 (0.53–3.80)
GE_45%_	22.80 ± 0.29	22.42 ± 0.47	0.95 (0.37–2.27)
SP (W)	1489.81 ± 235.89	1362.40 ± 138.91	0.66 (0.61–1.94)
SP (W/kg)	19.04 ± 2.22	15.93 ± 1.76	1.56 (0.09–3.00)
SP_10s_ (W)	1283.15 ± 179.18	1219.50 ± 120.99	0.42 (0.83–1.67)
SP_10s_ (W/kg)	16.39 ± 1.47	14.28 ± 1.70	1.32 (0.07–2.72)
PIP_45_ (%)	81.83 ± 1.91	77.16 ± 1.87	2.46 (0.73–4.19)

Data are presented as mean ± SD for elite and amateurs, Cohen’s d effect size (ES) and 95% confidence intervals (95% CI) are reported for each variable. ${\dot{V}O}_{2peak}$ , peak oxygen consumption; PPO, incremental test peak power output; GE_45%_, gross efficiency at 45% of PPO; SP, sprint peak power output; SP10s, sprint average (10s) power output; PIP_45%_, positive impulse percentage at 45% of PPO; ES, effect size; 95% CI, 95% confidence interval.

**FIGURE 1:**
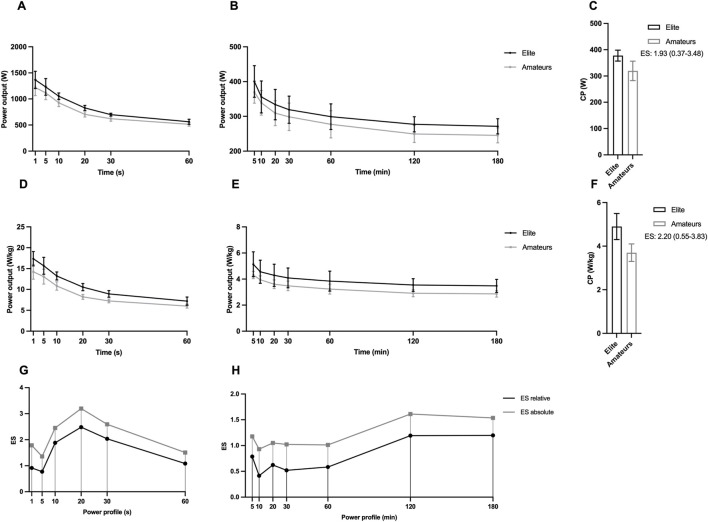
Absolute **(A–C)** and relative **(D–F)** power records and critical power (CP) data (mean ± SD) in elite and amateur cyclists. **(G,H)** represent the effect sizes for relative and absolute power profile data at the respective time point.

Power profile and CP obtained from seasonal best scores for elite and amateurs are reported in Figure 1. CP and Power scores were greater in elite especially when expressed in relative terms and with a constant trend across time intervals (CP absolute, ES: 1.93, 95% CI: 0.37–3.48; CP relative, ES: 2.20, 95% CI: 0.85–3.84 - power profile absolute, ES range: 0.41–2.48, 95% CI min-max range: 0.22–4.21; power profile relative, ES range: 0.93–3.19, 95% CI min-max range: 0.27–5.19).

**FIGURE 2:**
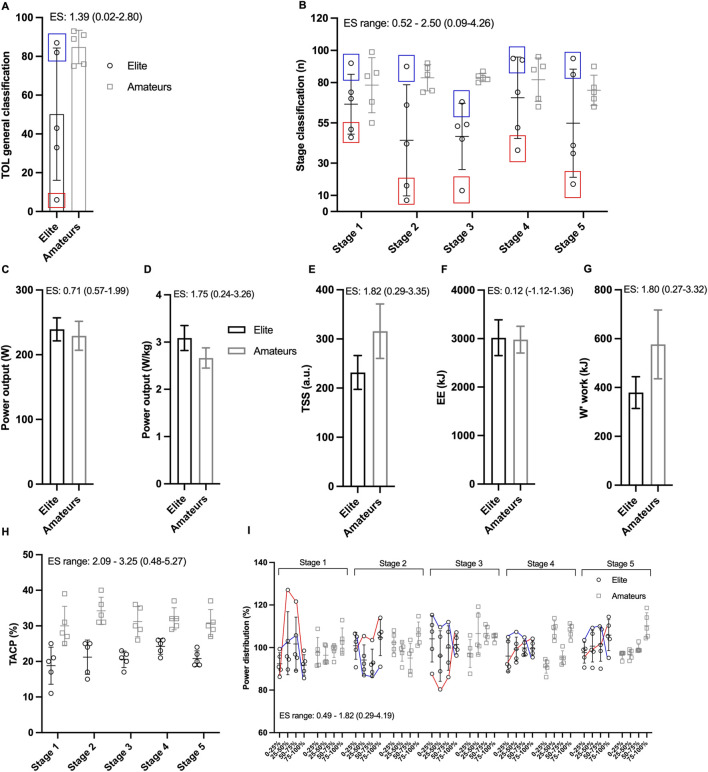
General classification **(A)** and stage-by-stage results **(B)** for elite and amateur cyclists in the Tour of Lithuania (TOL). Blue squares indicate elite domestiques, while red squares denote designated team leaders. **(C–F)** show power output (PO) **(C,D)**, training stress score (TSS) **(E)**, energy expenditure (EE) **(F)**, and W′ work **(G)** across the five stages. Time spent above critical power **(H)** and power output distribution **(I)** are also presented. In **(I)** each stage contains four bars representing different race segments (0%–25%, 25%–50%, 50%–75%, and 75%–100% of total stage duration) for both elite and amateur cyclists, expressed as a percentage of the stage mean power output. Stage-specific details: in Stage 1, the red line represents a breakaway attempt by an elite rider who spent nearly the entire stage at the front, while a teammate (blue line) tried to control the race till part of the breakaway was catch; in Stage 2, the red line highlights the power distribution of the elite team leader, who remained with the leading group and finished in the top 10, while in blue a domestique tried to control and work for the leader in the first phase and once the group was split in different part just tried to save energies for the next stage; in Stages 3 and 4, the blue line shows an elite domestique’s effort, characterized by pulling and closing gaps early before reducing effort in the final phase, while the red line represents the elite team’s sprinter, who conserved energy early before an all-out effort at the finish. Data are reported as mean ± SD.

Tour of Lithuania competition results and performance variables are illustrated in Figure 2. Power and video analysis highlighted the structured team roles in the elite squad, shaping their tactical approach (Figures 2A,B,G,H). Despite elite cyclists producing higher mean power, amateurs exhibited higher physiological load, reflected by greater training stress score, time spent above critical power, and accumulated W′ work (Figures 2C–F). These differences ultimately influenced individual stage performances and final TOL classification outcomes (Figures 2A,B). Specifically, elite cyclists consistently occupied leading positions in the general classification, with the team leader finishing within the top 10 overall and showing limited cumulative time gaps to the race winner (∼1-min final gap), while domestiques ranked lower in the GC. In contrast, amateur cyclists placed substantially further down the general classification (approximately within the 75th–95th percentiles from the top of the GC, with >9-min final time gaps), displaying larger cumulative deficits and minimal separation among teammates. At the stage level, elite cyclists achieved repeated top-10 finishes in sprint stages, whereas amateur riders predominantly finished within the main bunch without contesting top positions. All results are reported in aggregated form to preserve participant anonymity.

## 4 Discussion

Here, we present a descriptive comparison of laboratory and field performance variables between elite and amateur cyclists competing at the UCI Tour of Lithuania. Amateur cyclists spent more time above their critical power threshold during competitions, resulting in a more even power distribution across different race phases compared to elite cyclists. In contrast, elite cyclists exhibited a race role-specific effort distribution—for example, domestiques exerted themselves early in the race before dropping back, while team leaders conserved energy in the early phases and went all-out toward the end. This pattern was also reflected in the stage results: amateurs consistently finished in similar positions, often grouped together in a ‘survive-the-day’ mode. In contrast, elite results clearly distinguished team leaders, who secured top-10 positions in the general classification, from domestiques, who typically finished toward the lower end of the standings. Amateurs almost matched absolute power records to elite cyclists, but their higher body weight resulted in lower relative values. The high absolute power values likely contributed to the amateurs’ ability to successfully complete a high-level competition with relatively modest elevation gains (absolute power advantage) (Gregory et al., 2007). On the other hand, elite cyclists likely benefited not only from their higher aerobic capacity but also from their superior race experience—such as effective pacing and positioning within the bunch—and their tactical proficiency in understanding race dynamics and cooperating to achieve better results (e.g., Figure 2I). Their ability to execute role-specific strategies, with domestiques setting a hard pace early before dropping back and team leaders conserving energy for decisive moments, reflects a deeper strategic awareness. This approach likely enabled them to optimize effort distribution, react efficiently to race developments, and ultimately outperform amateurs, who lacked structured race execution and role-specific coordination.

The results may suggest that amateur cyclists’ lower aerobic capacity and reduced power-to-weight ratio (Table 1; Figure 2) remain potentially critical physical performance factors limiting their competitiveness in multi-stage races. Not only did the laboratory incremental test and competition average power outputs indicate a higher physical performance level in elite cyclists, but they also demonstrated superior efficiency in translating the energy turnover into external power output. The latter was evident by a moderate larger ES of the GE in elite cyclists compared to the amateurs. The superior physical performance level measured in the laboratory combined with seemingly greater experience in energy management during race, optimized pacing/drafting strategies and racing skills translates into superior performance (i.e., competition results) in the elite cyclists. Amateur cyclists showed higher total W’ work across the five stages, supporting the interpretation of greater physiological strain and less efficient pacing and energy use, rather than higher effective performance. These findings align with previous studies emphasizing the pivotal role of tactical race behavior and racing experience in efficiently utilizing energy, ultimately determining success in competitive cycling (Phillips and Hopkins, 2020; Cesanelli and Indaburu, 2021; Cesanelli et al., 2023; Cesanelli et al., 2024).

Amateur cyclists spent more time above their critical power threshold during the TOL stages, that may reflect an effort to keep up with the elite group, but likely resulted in earlier fatigue and reduced power output in the later segments of each stage. Analysis of power distribution across stages revealed a consistent pattern were elite cyclists strategically invested their energy based on predefined race roles (e.g., domestique, sprinter, or GC leader), indicating a more structured and tactical approach to competition. In contrast, no clear patterns emerged for amateur cyclists, whose power distribution appeared to be focused primarily on maintaining group cohesion and completing the competition (‘survive-the-day’ mode’). This may partly reflect a less defined team hierarchy or a reluctance among amateur riders to fully sacrifice their personal performance for team tactics, given the absence of professional incentives. These observations align with previous research, which highlights inefficient pacing, cornering costs, and suboptimal energy management as common challenges for less experienced cyclists (Cesanelli et al., 2023).

The ability of amateur cyclists to complete the TOL, despite their limitations, is noteworthy and highlights the continuous rise in performance levels within amateur cycling (Cesanelli et al., 2023; Cesanelli et al., 2024). Despite limited training time and the challenges of balancing multiple responsibilities, recent years have demonstrated the remarkable efforts of amateur athletes to push their limits (Ferrucci et al., 2021; Ferrucci et al., 2022). Advances in training methodologies and tools have supported the development of effective training sessions under restricted conditions. However, while these advancements have helped narrow the physical performance gap between elite and amateur cyclists, significant differences remain in race behavior, tactical experience, and driving skills. These aspects, along with physical performance limitations, represent critical obstacles for amateur cyclists.

### 4.1 Practical applications and limitations

This descriptive study highlights both the strengths and limitations of amateur cyclists striving to reach elite performance levels. Clear constraints on physical performance development in this population are dictated by differences in life priorities and training conditions. However, while we did not present here detailed data on training routines, it is reasonable to assume that amateur cyclists aim to make the most of their limited training time through the use of advanced methodologies and technologies. Still, the physical performance gap remains a primary limitation—one that is difficult to overcome without increased training volume, which is often constrained by work and life commitments in non-professional athletes. In contrast, performance-related aspects such as tactical decision-making, pacing strategies, energy management, and race-specific technical skills—though requiring time to develop—represent qualitative areas that may be improved with a qualitative approach, such as reviewing race footage, learning from experienced riders and coaches, and gaining exposure through more frequent racing. A major limitation of the present study is the lack of detailed data on training regimen differences between the two populations, which could provide deeper insights into performance gaps. Furthermore, despite following strict calibration procedures before each training session or competition, it was not possible to standardize the use of the same power meter model across all cyclists. Additionally, the results cannot be generalized beyond this cohort, as the study included only moderately high-level elite cyclists and competitive amateurs, and the race conditions were specific to the Tour of Lithuania, characterized by relatively flat roads and modest elevation gain, rather than the more demanding terrain encountered in other stage races. Furthermore, as is typical in sport teams, age, body morphology, composition, and performance characteristics were relatively homogeneous within each group; this should be considered when interpreting the results, particularly given the small cohort studied. Future studies should explore tailored training approaches that address the unique time constraints of amateur athletes, with a focus on closing the tactical and experiential gaps, and assess their impact on competition performance under similar conditions to those tested here.

### 4.2 Conclusions

This study highlights key differences in performance metrics and tactical behavior between elite and amateur cyclists during a multi-stage race. While amateur cyclists demonstrated moderately lower absolute power output and GE, their relative power output and aerobic capacity was largely reduced compared to elite cyclists that together with less efficient power distribution strategies limited their performance. Addressing these gaps through targeted training could improve their competitiveness in similar events.

## Data Availability

The original contributions presented in the study are included in the article/supplementary material, further inquiries can be directed to the corresponding author.
